# Perinatal Plasma Monocyte Chemotactic Protein-1 Concentrations in Intrauterine Growth Restriction

**DOI:** 10.1155/2007/65032

**Published:** 2007-11-18

**Authors:** Despina D. Briana, Maria Boutsikou, Stavroula Baka, George Papadopoulos, Dimitrios Gourgiotis, Karl Philipp Puchner, Dimitrios Hassiakos, Ariadne Malamitsi-Puchner

**Affiliations:** ^1^Second Department of Obstetrics and Gynecology, Athens University Medical School, 11528 Athens, Greece; ^2^Research Laboratories, Second Department of Pediatrics, Athens University Medical School, 11527 Athens, Greece

## Abstract

Monocyte-chemotactic-protein-1 (MCP-1) plays vital roles in immune response, angiogenesis, and pregnancy outcome. We investigated plasma MCP-1 concentrations in 40 mothers and their 20 intrauterine-growth-restricted (IUGR) and 20 appropriate-for-gestational-age (AGA) fetuses and neonates on postnatal days 1 (N1) and 4 (N4). Maternal and fetal MCP-1 concentrations were decreased (P<001 and P = .018, resp.), whereas N1 MCP-1 concentrations were elevated in IUGR group (P = .012). In both groups, fetal MCP-1 concentrations were lower compared to N1 and N4 ones (P = .045, P = .012, resp., for AGA, P < .001 in each case for IUGR). Reduced maternal and fetal MCP-1 concentrations in IUGR may reflect failure of trophoblast invasion, suggesting that down-regulation of MCP-1 may be involved in the pathogenesis of IUGR. Increased MCP-1 concentrations in IUGR neonates and higher postnatal ones in all infants may be attributed to gradual initiation of ex utero angiogenesis, which is possibly enhanced in IUGR.

## 1. INTRODUCTION

Evidence is accumulating that maintenance of a normal pregnancy is dependent on a delicate interaction between the endocrine and immune systems [1]. Disturbance of this balance can result in a wide range of abnormalities including intrauterine growth restriction (IUGR) [2].

Cytokines, the local effectors of the immune system, are increasingly thought to play a key role during pregnancy [1, 3]. Chemokines, a subset of cytokines, specific in their ability to attract and activate immune cells, are thought to play pivotal roles in the immune recognition, maintenance of pregnancy, and parturition [4]. In this respect, unbalanced chemokine expression may contribute to defective placentation and pregnancy failures [3].

Monocyte chemotactic protein (MCP)-1 is a 76-amino acid **β** chemokine, which is secreted by a wide range of cell types, including monocytes [5], macrophages [6], lymphocytes [7], and endothelial cells [8]. It primarily serves as a chemotactic factor that attracts and activates monocytes/macrophages into cites of inflammation, by inducing leukocyte-endothelial cell adhesion and promoting transendothelial migration [6, 9]. Recently, a number of other functions have been ascribed to MCP-1, including that of an angiogenic factor [10, 11]. Moreover, MCP-1 expression has been demonstrated in maternal gestational tissues, such as decidua and myometrium, as well as in embryonically-derived ones, including chorion and placenta [4]. MCP-1 production increases during normal pregnancy, and even more during labor, suggesting that MCP-1 may play a role, which is more important as pregnancy advances, by locally modulating the immune system at the level of peripheral leukocytes [1]. However, the perinatal expression of MCP-1 has not been, up to the present, investigated in IUGR pregnancies.

In this respect, this study was based on the hypothesis that circulating concentrations of MCP-1 might differ between IUGR cases and appropriate for gestational age (AGA) controls, since the former are characterized by abnormal immune responses, leading to inappropriate cytokine secretion [12, 13], and impaired angiogenesis [14–17]. Therefore, we aimed to evaluate and compare, for the first time to our knowledge, plasma MCP-1 concentrations in IUGR and AGA mother/infant pairs at crucial perinatal time points and investigate the association of its circulating concentrations with gender, parity, mode of delivery and, adjusted birth weight (customized centiles).

## 2. MATERIALS AND METHODS

The Ethics Committee of our teaching hospital approved the study protocol. All included mothers provided signed informed consent before recruitment. Forty parturients giving consecutively birth, either to 20 AGA or 20 asymmetric IUGR full-term singleton infants (birth weight ≤ 3rd customized centile), were included in the study. The gestation-related optimal weight computer-generated program [18, 19] was used to calculate the customized centile for each pregnancy, taking into consideration significant determinants of birth weight, such as maternal height, booking weight, ethnic group, parity, gestational age, and gender [18]. Gestational age was estimated using the date of the last menstrual period and early antenatal ultrasound. Birth weight was measured with an electronic scale.

Nine of the 20 mothers with IUGR offspring presented with preeclampsia [20]. The remaining 11 mothers presented with pregnancy-induced hypertension and in addition, suffered from iron-deficient anemia (3 cases), gestational diabetes mellitus (2 cases), hypothyroidism (3 cases), extreme obesity (2 cases), and cardiac arrhythmias (1 case). Five of the above women were smoking >10 cigarettes/day during the whole duration of pregnancy.

Doppler studies were performed in the IUGR group every 10–15 days, starting from the 32nd gestational week. During each Doppler velocimetry evaluation, three consecutive measurements of the pulsatility index (PI) of the studied vessel (uterine, umbilical, cerebral arteries) were done, and the mean value was recorded. Concerning uterine and umbilical arteries [21, 22], mean PI values were progressively found to be in the upper physiological limits for the corresponding gestational age in 13 cases (ranging between the 90th and the 95th percentile) while in the remaining seven cases PI values showed increased impedance to flow, being above the 95th percentile for gestational age. Regarding middle cerebral arteries [23], Doppler studies showed resistance to be in the lower physiological limits for gestational age, indicating the initiation of blood flow redistribution process, in order to spare vital organs (brain, heart, and adrenals). Nevertheless, amniotic fluid was diminished in all IUGR cases. For the evaluation of the amniotic fluid, the largest fluid column on the vertical plane was assessed and was defined as diminished, if <2 cm. Placental weights were reduced ranging from 255 to 400 g.

In the AGA group, mothers were healthy and were either nonsmokers or abstained from smoking during pregnancy. Placentas were normal in appearance and weight.

Tests for congenital infections were negative in all women of both groups, and their offspring had no symptoms of intrauterine infection or signs of genetic syndromes. One- and five-minute Apgar scores were in all cases and controls ≥8. All neonates were breastfed. Demographic data of participating subjects are listed in Table 1.

Blood was collected in pyrogen-free tubes from the following: (i) the mothers during the first stage of labor, or before receiving anesthesia in cases of elective caesarean section; (ii) the umbilical cords after double clamping, reflecting fetal state; and (iii) the neonates before feeding on postpartum days 1 (N1) and 4 (N4), characterizing transition and stabilization to extrauterine life, respectively. Plasma was separated by centrifugation and was kept frozen at $-80^{\circ }\text{C}$ until assay.

The determination of plasma MCP-1 concentrations was performed by ELISA (MCP-1 (Human) ELISA Kit, HBT (HyCult Biotechnology b.v) UDEN, the Netherlands). The minimum detectable concentration, intra- and interassay coefficients of variation were <10 pg/mL, 6.2% and 7.7%, respectively.

## 3. STATISTICAL ANALYSIS

MCP-1 data were normally distributed (Kolmogorov-Smirnov test); thus, parametric tests (Anova for repeated measures, paired samples t-test with Bonferroni correction for multiple comparisons) were applied in the analysis. Spearman's or Pearson's correlation coefficients, where appropriate, were used to detect any positive or negative correlations. *P* < .05 was considered statistically significant.

## 4. RESULTS

**Figure 1:**
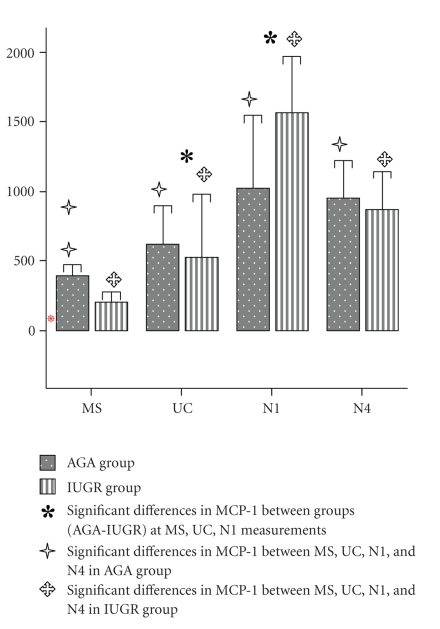
MCP-1 concentrations in the plasma of mothers (MS), fetuses (UC), and neonates on day 1 (N1) and 4 (N4) postpartum in appropriate for gestational age (AGA) and intrauterine growth restricted (IUGR) groups. Bars represent the mean values and error bars one SD of the mean.

Determined mean (95% confidence intervals (CI)) values of circulating MCP-1 concentrations in both groups are shown in Figure 1.

Maternal and fetal MCP-1 concentrations were significantly decreased in the IUGR compared to the AGA group after adjusting for multiple comparisons (*b*: 167.018, 95% CI: 114.511–219.525, *P* < .001 and *b*: 216.322, 95% CI: 40.423–392.221, *P* = .018, resp.). On the contrary, N1 MCP-1 concentrations were significantly elevated in the IUGR compared to the AGA group after adjusting for multiple comparisons ((*b*: −467.934, 95% CI: −824.383−(−111.484), *P* = .012).

In the AGA group, maternal MCP-1 concentrations were significantly lower compared to fetal, N1 and N4 ones (*P* = .019, *P* = .001, and *P* < .001, resp.) while fetal MCP-1 concentrations were significantly lower compared to N1 and N4 ones (*P* = .045 and *P* = .012, resp.).

In the IUGR group, maternal MCP-1 concentrations were significantly lower compared to fetal, N1 and N4 ones (*P* = .003, *P* < .001 and *P* < .001, resp.) while fetal MCP-1 concentrations were significantly lower compared to N1 and N4 ones (*P* < .001 in each case). Finally, in each (IUGR or AGA) group, the effect of gender, mode of delivery, and parity on plasma MCP-1 concentrations was not significant.

## 5. DISCUSSION

During gestation, inflammatory cytokines, occasionally more abundant than growth-promoting ones, lead via direct or indirect effects to IUGR [24]. In this respect, many recent studies point to altered cytokine pattern in IUGR pregnancies, documenting up- or downregulation of several cytokines in amniotic fluid, as well as maternal and fetal circulation [2, 12, 25–30]. Thus, elevated TNF-alpha and reduced interleukin (IL)-1 beta amniotic fluid concentrations [2], as well as enhanced placental TNF-alpha secretion [31], have been implicated in vasoconstriction of the feto-placental vascular bed, resulting in IUGR. Furthermore, elevated maternal TNF-alpha, IL-6 [2, 25], IL-8 [25], and macrophage colony-stimulating factor [26] concentrations have been observed in IUGR due to placental insufficiency. On the other hand, reduced placental IL-6 production has been associated with placental ischemia and failure of fetal growth in pregnancy-induced hypertension [27]. Similarly, reduced placental IL-10 expression has been involved in the pathogenesis of IUGR [32].

In line with these reports, the results of the present study indicate that circulating maternal and fetal MPC-1 concentrations are significantly lower in IUGR cases compared to AGA controls. Successful placentation involves the development of a highflow uteroplacental circulation, which is established by trophoblast invasion and transformation of the maternal intramyometrial portion of the spiral arterioles [33]. Asymmetric fetal growth restriction is attributed to reduced placental blood flow with subsequent impaired fetomaternal exchange of substrates, a process probably initiated by unsuccessful transformation of uteroplacental spiral arteries [34]. In this respect, Brosens et al. showed that the expected physiological changes in pregnancy are restricted in the decidual segments of the uteroplacental arteries in all hypertensive patients with IUGR offspring [35]. This failure of placentation has been attributed to inadequate decidual cellular immunity, limiting trophoblastic invasion [34]. Thus, it has been hypothesized that placental insufficiency in IUGR could be caused by an immunological phenomenon [12].

MCP-1, abundantly produced during gestation by endometrial, myometrial, placental, and trophoblast cell types [4], has been reported to play specific roles in endometrial angiogenesis, apoptosis, proliferation, and differentiation [3]. Furthermore, MCP-1 plays an essential role in maintaining pregnancy [1, 4]. Based on these facts, we speculate that MCP-1 may regulate trophoblast invasion into the placental bed and may influence placental development and function by acting via decidual and fetal macrophages, as previously stated for macrophage colony-stimulating factor [36]. Thus, the lower maternal and fetal MCP-1 concentrations in our IUGR cohort may indicate failure of trophoblast invasion, suggesting that downregulation of MCP-1 may be involved in the pathogenesis of IUGR.

Controversial data exist in the literature, regarding circulating MCP-1 concentrations in preeclampsia. Thus, a recent study demonstrated significantly elevated MCP-1 concentrations in preeclamptic patients [37], whereas other researchers found no significant differences in plasma MCP-1 concentrations between preeclamptic and normal pregnant women [38]. In the former study [37], it has been hypothesized that, in preeclampsia, the damaged endothelial cells and/or infiltrated macrophages may produce MPC-1, which probably promotes extension of the lesions. A possible explanation for our contradictory finding may relate to the fact that more than half of the women with IUGR offspring in our study presented with pregnancy-induced hypertension, which is not associated with endothelial dysfunction [39] and thus, with probably higher MCP-1 production.

On the other hand, our study demonstrated increased circulating MCP-1 concentrations in IUGR neonates compared to AGA controls, as well as higher postnatal concentrations in all included infants. MCP-1 has been reported to directly promote angiogenesis, as a consequence of leukocyte infiltration, or growth factor expression [40]. Therefore, above findings may be attributed to a gradual initiation of ex utero angiogenesis, a prerequisite for tissue growth and development [41], which is possibly enhanced in IUGR cases, due to a potential initiation of the catch-up growth process [42]. In addition, the reported higher fetal MCP-1 concentrations compared to respective maternal ones may be attributed to the fact that, in late gestation, MCP-1 is predominantly released by embryonically derived tissues, including the chorion and the placenta [4].

Finally, gender, parity, and the mode of delivery did not prove to have a significant effect on circulating MCP-1 concentrations in this study. Relatively, a recent report documented significantly higher MCP-1 concentrations in the placentas of primigravidas [24]. Furthermore, amniotic fluid concentrations of MCP-1 reportedly increase during spontaneous labour, suggesting its potential role in this process [43].

In conclusion, the reduced maternal and fetal MCP-1 concentrations in our IUGR cohort may reflect failure of trophoblast invasion, suggesting that downregulation of MCP-1 may be involved in the pathogenesis of IUGR. On the other hand, the increased MCP-1 concentrations in IUGR neonates, as well as the higher postnatal concentrations in all included infants may be attributed to a gradual initiation of ex utero angiogenesis, which is possibly enhanced in IUGR cases. Additional studies are required to elucidate the physiological role of this chemokine in both normal and complicated pregnancies.

## Figures and Tables

**Table 1:** Demographic data for AGA and IUGR neonates and their mothers.

	AGA	IUGR	*P*
	Mean (SD)	Mean (SD)	
Birthweight (g)	3356 (223)	2342 (229)	< .001
Birthweight centile	65.4 (12.6)	1.5 (1.5)	< .001
Gestational age (weeks)	38.4 (1.0)	37.8 (1.0)	< .024
Gender			NS
* *Male	11 (55%)	11 (55%)	
* *Female	9 (45%)	9 (45%)	
Maternal age (years)	28 (4.0)	31 (5.0)	NS
Parity			NS
* *Primigravida	10 (50%)	11 (55%)	
* *Other	10 (50%)	9 (45%)	
Mode of delivery			< .013
* *Vaginal	13 (65%)	8 (40%)	
* *Cesarean section	7 (35%)	12 (60%)	

## References

[1] Denison FC, Kelly RW, Calder AA (1997). Differential secretion of chemokines from peripheral blood in pregnant compared with non-pregnant women. *Journal of Reproductive Immunology*.

[2] Stallmach T, Hebisch G, Joller-Jemelka HI, Orban P, Schwaller J, Engelmann M (1995). Cytokine production and visualized effects in the feto-maternal unit: quantitative and topographic data on
cytokines during intrauterine disease. *Laboratory Investigation*.

[3] Kayisli UA, Mahutte NG, Arici A (2002). Uterine chemokines in reproductive physiology and pathology. *American Journal of Reproductive Immunology*.

[4] Denison FC, Kelly RW, Calder AA, Riley SC (1998). Cytokine secretion by human fetal membranes, decidua and placenta at term. *Human Reproduction*.

[5] Yoshimura T, Robinson EA, Tanaka S, Appella E, Kuratsu J-I, Leonard EJ (1989). Purification and amino acid analysis of two human glioma-derived monocyte chemoattractants. *Journal of Experimental Medicine*.

[6] Brieland JK, Jones ML, Flory CM (1993). Expression of monocyte chemoattractant protein-1 (MCP-1) by rat alveolar macrophages during chronic lung injury. *American Journal of Respiratory Cell and Molecular Biology*.

[7] Yoshimura T, Yuhki N, Moore SK, Appella E, Lerman MI, Leonard EJ (1989). Human monocyte chemoattractant protein-1 (MCP-1). Full length cDNA cloning, expression in mitogen-stimulated blood mononuclear leukocytes, and sequence similarity to mouse competence gene JE. *FEBS Letters*.

[8] Sica A, Wang JM, Colotta F (1990). Monocyte chemotactic and activating factor gene expression induced in endothelial cells
by IL-1 and tumor necrosis factor. *Journal of Immunology*.

[9] Proost P, Wuyts A, van Damme J (1996). Human monocyte chemotactic proteins-2 and -3: structural and functional comparison with MCP-1. *Journal of Leukocyte Biology*.

[10] Salcedo R, Ponce ML, Young HA (2000). Human endothelial cells express CCR2 and respond to MCP-1: direct role of MCP-1 in angiogenesis
and tumor progression. *Blood*.

[11] Kim MY, Byeon CW, Hong KH, Han KH, Jeong S (2005). Inhibition of the angiogenesis by the MCP-1 (monocyte chemoattractant protein-1) binding peptide. *FEBS Letters*.

[12] Bartha JL, Romero-Carmona R, Comino-Delgado R (2003). Inflammatory cytokines in intrauterine growth retardation. *Acta Obstetricia et Gynecologica Scandinavica*.

[13] Bartha JL, Comino-Delgado R (1999). Lymphocyte subpopulations in intrauterine growth retardation in women with or without previous pregnancies. *European Journal of Obstetrics Gynecology & Reproductive Biology*.

[14] Regnault TRH, de Vrijer B, Galan HL (2003). The relationship between transplacental ${\text{O}}_{2}$ diffusion and placental expression of PIGF, VEGF and their
receptors in a placental insufficiency model of fetal growth restriction. *Journal of Physiology*.

[15] Malamitsi-Puchner A, Boutsikou T, Economou E (2005). The role of the anti-angiogenic factor endostatin in intrauterine growth restriction. *Journal of the Society for Gynecologic Investigation*.

[16] Malamitsi-Puchner A, Boutsikou T, Economou E (2005). Vascular endothelial growth factor and placenta growth factor in intrauterine growth-restricted fetuses and neonates. *Mediators of Inflammation*.

[17] Malamitsi-Puchner A, Boutsikou T, Economou E (2006). Angiopoietin-2 in the perinatal period and the role of intrauterine growth restriction. *Acta Obstetricia et Gynecologica Scandinavica*.

[18] Gardosi J, Chang A, Kalyan B, Sahota D, Symonds EM (1992). Customised antenatal growth charts. *The Lancet*.

[19] Gardosi J, Mongelli M, Wilcox M, Chang A (1995). An adjustable fetal weight standard. *Ultrasound in Obstetrics & Gynecology*.

[20] Beroyz G, Casale R, Farreiros A (1994). CLASP: a randomised trial of low-dose aspirin for the prevention and treatment of pre-eclampsia among
9364 pregnant women. *The Lancet*.

[21] Kaminopetros P, Higueras MT, Nicolaides KH (1991). Doppler study of uterine artery blood flow: comparison of findings in the first and second trimesters of pregnancy. *Fetal Diagnosis and Therapy*.

[22] Acharya G, Wilsgaard T, Berntsen GKR, Maltau JM, Kiserud T (2005). Reference ranges for serial measurements of umbilical artery Doppler indices in the second half of pregnancy. *American Journal of Obstetrics and Gynecology*.

[23] Baschat AA, Galan HL, Bhide A (2006). Doppler and biophysical assessment in growth restricted fetuses: distribution of test results. *Ultrasound in Obstetrics and Gynecology*.

[24] Bouyou-Akotet MK, Kombila M, Kremsner PG, Mavoungou E (2004). Cytokine profiles in peripheral, placental and cord blood in pregnant women from an area endemic for
Plasmodium falciparum. *European Cytokine Network*.

[25] Stallmach T, Hebisch G, Joller H, Kolditz P, Engelmann M (1995). Expression pattern of cytokines in the different compartment of the feto-maternal unit under various conditions. *Reproduction, Fertility and Development*.

[26] Hayashi M, Ohkura T (2002). Elevated levels of serum macrophage colony-stimulating factor in normotensive pregnancies
complicated by intrauterine fetal growth restriction. *Experimental Hematology*.

[27] Yin C, Tian Y, Zheng Y (1998). Study on relationship between detection of interleukin-6 and its mRNA and pregnancy induced hypertension. *Zhonghua Fu Chan Ke Za Zhi*.

[28] Amu S, Hahn-Zoric M, Malik A (2006). Cytokines in the placenta of Pakistani newborns with and without intrauterine growth retardation. *Pediatric Research*.

[29] Street ME, Seghini P, Feini S (2006). Changes in interleukin-6 and IGF system and their relationships in placenta and cord
blood in newborns with fetal growth restriction compared with controls. *European Journal of Endocrinology*.

[30] Ødegård RA, Vatten LJ, Nilsen ST, Salvesen KÅ, Vefring H, Austgulen R (2001). Umbilical cord plasma interleukin-6 and fetal growth restriction in preeclampsia: a prospective study in Norway. *Obstetrics & Gynecology*.

[31] Holcberg G, Huleihel M, Sapir O (2001). Increased production of tumor necrosis factor-α TNF-α by IUGR human placentae. *European Journal of Obstetrics Gynecology & Reproductive Biology*.

[32] Hahn-Zoric M, Hagberg H, Kjellmer I, Ellis J, Wennergren M, Hanson LÅ (2002). Aberrations in placental cytokine mRNA related to intrauterine growth retardation. *Pediatric Research*.

[33] Genbacev O, Joslin R, Damsky CH, Polliotti BM, Fisher SJ (1996). Hypoxia alters early gestation human cytotrophoblast differentiation/invasion in vitro and models
the placental defects that occur in preeclampsia. *Journal of Clinical Investigation*.

[34] Khong TY, De Wolf F, Robertson WB, Brosens I (1986). Inadequate maternal vascular response to placentation in pregnancies complicated by
pre-eclampsia and by small-for-gestational age infants. *British Journal of Obstetrics and Gynaecology*.

[35] Brosens I, Dixon HG, Robertson WB (1977). Fetal growth retardation and the arteries of the placental bed. *British Journal of Obstetrics and Gynaecology*.

[36] Jokhi PP, Chumbley G, King A, Gardner L, Loke YW (1993). Expression of the colony stimulating factor-1 receptor (c-fms product) by cells at the human uteroplacental interface. *Laboratory Investigation*.

[37] Katabuchi H, Yih S, Ohba T (2003). Characterization of macrophages in the decidual atherotic spiral artery with 
special reference to the cytology of foam cells. *Medical Electron Microscopy*.

[38] Jonsson Y, Rubèr M, Matthiesen L, Berg G (2006). Cytokine mapping of sera from women with preeclampsia and normal pregnancies. *Journal of Reproductive Immunology*.

[39] Bosio PM, McKenna PJ, Conroy R, O'Herlihy C (1999). Maternal central hemodynamics in hypertensive disorders of pregnancy. *Obstetrics and Gynecology*.

[40] Bernardini G, Ribatti D, Spinetti G (2003). Analysis of the role of chemokines in angiogenesis. *Journal of Immunological Methods*.

[41] Jackson JR, Seed MP, Kircher CH, Willoughby DA, Winkler JD (1997). The codependence of angiogenesis and chronic inflammation. *FASEB Journal*.

[42] Chakraborty S, Joseph DV, Bankart MJ, Petersen SA, Wailoo MP (2007). Fetal growth restriction: relation to growth and obesity at the age of 9 years. *Archives of Disease in Childhood. Fetal and Neonatal Edition*.

[43] Esplin MS, Romero R, Chaiworapongsa T (2005). Monocyte chemotactic protein-1 is increased in the amniotic fluid of women who deliver preterm
in the presence or absence of intra-amniotic infection. *Journal of Maternal-Fetal & Neonatal Medicine*.

